# Study of Minor Physical Anomalies in Complete Nuclear Mexican Families. Evidence of Neurodevelopmental Problems in Schizophrenia

**DOI:** 10.1371/journal.pone.0117080

**Published:** 2015-01-22

**Authors:** Félix Ambrosio-Gallardo, Carlos Cruz-Fuentes, Gerhard Heinze-Martin, Jorge Caraveo-Anduaga, José Cortés-Sotres

**Affiliations:** 1 Hospital Psiquiátrico Fray Bernardino Álvarez, Mexico City, Mexico; 2 Departamento de Genética, Instituto Nacional de Psiquiatría Ramón de la Fuente Muñiz, México City, Mexico; 3 Departamento de Psiquiatría y Salud Mental, Facultad de Medicina, Universidad Autónoma de México, Mexico City, Mexico; 4 Investigaciones Epidemiológicas y Sociales, Instituto Nacional de Psiquiatría Ramón de la Fuente Muñiz, México City, Mexico; 5 Departamento de Apoyo Académico, Instituto Nacional de Psiquiatría Ramón de la Fuente Muñiz, México City, Mexico; Maastricht University, NETHERLANDS

## Abstract

**Introduction:**

Minor physical anomalies (MPA) are dysmorphic features that reflect deviations in early development, are morphological variants that appear during the first trimester of pregnancy and could be used as a marker of disease risk in susceptible people. The literature agrees that the number of MPA is higher in patients with schizophrenia compared with their relatives and healthy subjects. The purpose of this study is to compare the MPA, assessed using the Gourion Scale, in complete nuclear families (families with a member with schizophrenia and control families) by determining the MPA mean, concordance and heritability for the total score on the MPA Gourion Scale for each anomaly.

**Method:**

The sample consisted of 60 families with at least one schizophrenic patient (284 members) and 61 control families (249 members). Results: The mean total score for the scale was 5.72 ± 2.3 MPA in the case of families with at least one schizophrenic patient and 1.8 ± 4.46 MPA for control families. The average for families of patients without considering the patient in the analysis was 5.59 ± 2.3 MPA; for patients, the mean was 6.14 ± 2.4 MPA. In the analysis by anomaly differences were found only in eleven anomalies found no evidence of heritability or concordance.

**Conclusions:**

MPA occur more frequently in patients, but a pattern of low consistency between them persists. It is concluded that MPA could be a marker of neurodevelopmental problems, but it is not suitable to consider them a Gourion scale as endophenotype.

## Introduction

Among the various theories on the etiology of schizophrenia, one of the most accepted models is to consider it a neurodevelopmental disorder. The neurodevelopmental hypothesis of schizophrenia postulates that schizophrenia is a developmental disorder associated with a brain defect resulting from genetic events or abnormal epigenetics and is influenced by a diverse set of environmental events, both prenatal and postnatal. Support for this neurodevelopmental hypothesis is abundant [1–4], although there are some authors who are critical of the position [5–6]. The evidence supporting the neurodevelopmental hypothesis is summarized by McClure and Lieberman [7]. The literature reports six categories of anomalies associated with schizophrenia: major congenital anomalies, structural brain abnormalities, medical comorbidities, prenatal exposure to infections, maternal obstetric complications and minor physical anomalies (MPA) (the purpose of this study).

MPA represent dysmorphic features reflecting “subtle” deviations in the early development of individual structures in the head, eyes, ears, mouth, hands and feet. Once formed, MPA persist into adulthood and can be evaluated reliably through the visual examination of a particular region of the body [8], they are morphological variants appearing during the first or second trimester of gestation without presenting a significant functional or cosmetic impact and could be used as a risk marker for disease in susceptible individuals [9–10].

The listings of MPA have been developed from 1967 to the present; Goldfarb and Botstein [11] developed a list of 21 anomalies. Waldrop [12], based on the anomalies listed by Goldfarb, constructed a scale with 18 anomalies, and this scale and its modifications have been used in the study of schizophrenia.

A meta-analysis by Weinberg [13], conducted before the numerous studies that report an increase in the frequency of minor physical anomalies (MPA) in individuals with schizophrenia compared to healthy controls, found that the studies reviewed varied considerably with respect to the magnitude of difference between cases and controls and of the topographical distribution of the anomalies. The magnitude of the pooled effect size of total MPA scores was d = 1.13 (p <0,001), indicating that MPA are significantly present in individuals with schizophrenia. Another meta-analysis by Xu [14] in 2011, examining 14 studies, found an effect size between patients and healthy controls of d = 0.95. Six studies including patients and their first-degree family members showed an average effect size of d = 0.45, but a small and insignificant effect size, d = 0.32, between the family members of patients and healthy controls. These results are consistent with the idea that MPA may represent a putative endophenotype for schizophrenia. Seven other publications that show an increased number of MPA between patients with schizophrenia and control subjects were not included in the meta-analysis [15–21]. Three other items not included in the meta-analysis compare patients with family members but only report differences found for each anomaly, ranging from three to seven MPA without consistency between publications [22–24]. There is only one longitudinal study, by Schiffman (2002), who studied a cohort consisting of Danish children born between 1959 and 1961 evaluated for MPA. Of the subjects with three or more MPA in infancy, 8.3% developed schizophrenia and 16.5% developed another psychiatric disorder. It is concluded that MPA are a risk factor for schizophrenia [25].

The literature concurs that there is an excess of MPA in schizophrenic patients compared with normal subjects. In the reviewed studies, the comparison of MPA with family members is not very systematic, and in several of them, the number of family members studied is smaller than the number of patients. MPA comparisons between patients and their parents or the nuclear complete family have not been studied. The purpose of this study is to compare MPA between complete nuclear families (families with a member with schizophrenia and control families), determining the familial aggregation (concordance and heritability) of MPA. Evidence of familial aggregation was considered to exist when there were differences between members of the control families and case families that persist when the analysis of the patient in the case families is removed and when there are no differences between the first-degree family members of the patient and the patient, either as a scale or for each individual MPA.

## Methods

### Participants

This study was approved by Ethical Committee of the Hospital Psiquiatrico Fray Bernardino Alvarez" and all participants signed informed consent. Family groups were chosen from the patients and family members of the “Fray Bernardino Álvarez” Psychiatric Hospital in Mexico City, F.D., with prior approval from the Institutional Ethics Committee, named “Comite de Etica e Investigación del Hospital Psiquiatrico Fray Bernardino Alvarez”.

Patients with schizophrenia according to the DSM-IV-TR, male or female, with both parents of Mexican population and with first-degree family members who agree to participate in the study. Patients whose condition prevented them from participating in the study were excluded, this condition was to the patient may be agitated, the evaluation of minor physical abnormalities is only by observation. Family members from both case families and control families were included when they had no personal history of psychiatric disease, male or female, between 18 to 65 years. Family members whose condition prevented them from participating in the study were excluded. All the families (cases and controls) should be nuclear, consisting of a father, mother and at least two offspring, and, in cases, families with at least one offspring with schizophrenia. All participants signed informed consent. Family groups were chosen from the patients and family members of the “Fray Bernardino Álvarez” Psychiatric Hospital in Mexico City, F.D., with prior approval from the institutional Ethics Committee.

Sixty-two families with at least one member diagnosed with schizophrenia and 61 control families all participated. The total sample consisted of 533 subjects, 284 in the group of families with one schizophrenic member, 66 of whom had a diagnosis of schizophrenia: 62 probands, 2 fathers and 2 mothers; 218 are family members of the probands, of whom 60 are fathers, 60 mothers, 53 unaffected brothers and 74 unaffected sisters. The control group families consisted of 249 subjects: 61 fathers, 61 mothers, 53 brothers and 74 sisters. In Table 1, the distribution by kinship and age is presented. The evolution time is 11.9±10.1years for male patients and 11.1±14.3 years for female patients.

**Table 1:** Sample distribution by group, kinship and age.

	Control Families				Case Families			
			Age				Age	
Kinship	n	%	Mean	SD	n	%	Mean	SD
Father with schizophrenia					2	0.4	65.5	2.1
without schizophrenia	61	11.4	57.1	9.6	60	11.3	59.7	12.1
Mother with schizophrenia					2	0.4	57.0	4.2
without schizophrenia	61	11.4	54.5	9.2	60	11.3	57.3	10.3
Brother with schizophrenia					45	8.4	31.1	8.5
without schizophrenia	53	9.9	25.7	7.6	39	7.3	31.3	9.9
Sister with schizophrenia					17	3.2	30.5	7.4
without schizophrenia	74	13.9	27.1	9.1	59	11.1	31.7	10.7
Total	249	46.7	40.9	17.2	284	53.3	43.2	17.0

SD Standard Deviation

### Evaluation Instruments

**M.I.N.I.** The M.I.N.I. (Mini-International Neuropsychiatric Interview) is a brief and highly structured interview on the major psychiatric disorders of the ICD-10 and DSM-IV [26]. The M.I.N.I. was used in its computerized version [27]. This interview was conducted using the application guidelines of the Diagnostic Interview for Genetic Studies (DIGS) [28].

**Modified Waldrop Scale.** This scale is a modification of the Waldrop Scale developed by Gourion. [29] The selection of anomalies was based on the frequency and reliability of the assessment of each anomaly. This scale includes 41 MPA: 17 based on Waldrop (1968), 20 MPA based on Ismail (1998) and four based on deletions of 22q11. All the items were scored as 0 (absent) or 1 (present). The inter-rater reliability, evaluated using the intraclass correlation coefficient, is 0.97.

A document was developed to compile the clinical and demographic data.

### Procedure

The sample was obtained from the “Fray Bernardino Álvarez” Psychiatric Hospital in Mexico City, F.D. Consent was obtained from the families to proceed with application of the M.I.N.I. interview to confirm the diagnosis of schizophrenia in the probands and to rule out any diagnosis of Axis I or Axis II in participating family members. Once diagnosis was established, the application of the Gourion MPA Scale and a questionnaire on sociodemographic data for all study participants was performed.

### Statistical analysis

To compare the total MPA, an overall Simple ANOVA was used. The association for each MPA was determined by Fisher’s exact test. Concordance was assessed by the percentage of the selected pair that was consistent with the presence of the anomaly. Heritability was calculated for the total Gourion Scale by the Mid Parent method [30], which consists of determining the regression coefficient for each dimensional measurement where the values of the parents predict the values of the offspring.

## Results

The members of the case families showed an increased number of MPA compared with the members of the control families. The averages obtained were 5.72±2.3 anomalies and 4.46±1.8 anomalies [F(1,531) = 47.93, p<0.001]; the members of the case families excluding patients also presented a higher number of MPA than the members of the control families, and the averages obtained were 5.59±2.3 anomalies and 4.46±1.8 anomalis [F(1,465) = 35.48, p<0.001]. The patients showed no difference when compared with their family members. This pattern is present in all areas except the ears. Table 2.

**Table 2:** Mean differences of Minor Physical Anomalies for Gourion’s Scale Total.

Group	n	Mean	SD	Significance
Total sample	533	5.13	2.2	
Control family members	249	4.46	1.8	F(1,531) = 47.93, p<0.001
Case family members	284	5.72	2.3	
Schizophrenic patients	66	6.14	2.4	F(1,282) = 2.86, p = 0.092
First grade relatives of schizophrenic patients	218	5.59	2.3	
Control family members	249	4.46	1.8	F(1,465) = 35.48, p<0.001
First grade relatives of schizophrenic patients	218	5.59	2.3	

SD Standard Deviation

The Gourion MPA scale distinguishes between families with a schizophrenic member and the control families for a cutoff of 7, with a specificity of 94.8%, a sensitivity of 23.2%, a positive predictive value of 83.5% and a negative predictive value of 52.0%. The Gourion MPA scale distinguishes between patients with schizophrenia and their first-degree family members for a cutoff of 9, with a specificity of 95.9%, a sensitivity of 7.6%, a positive predictive value of 35.7% and a negative predictive value of 77.4%.

In the analysis of individual MPA, eleven presented the expected pattern for familial aggregation: clinodactyly, fine electric hair, Big gap between first and second toes (sandal gap), the overlap of the toes, hyperconvex nails on the hands, the basis of an abnormally large nose, thin upper lip, ear lobes adherent, significant asymmetry of the face, fused eyebrows, epicanthus and an abnormally short toe. Table 3.

**Table 3:** Minor physical anomalies proportions comparison between groups.

Anomaly	Control Families (CoF)	Case Families (CaF)	Case Families without Patient (CaF w/P)	Patient(P)	Fisher exact Test	Fisher exact Test	Fisher exact Test
	% (n)	% (n)	% (n)	% (n)	CoF vs CaF	CoF vs CaF w/P	P vs CaF w/P
Curved fifth finger (Clinodactyly)	57.4 (143)	77.1 (219)	75.7 (165)	81.8 (54)	p<0.001	p<0.001	0.322
Fine electric hair	41.0 (102)	60.6 (172)	66.5 (145)	40.9 (27)	p<0.001	p<0.001	p<0.001
Big gap between first and second toes (sandal gap)	17.3 (43)	45.8 (130)	43.1 (94)	54.5 (36)	p<0.001	p<0.001	0.121
Overlapping toes (any pair)	62.2 (155)	44.7 (127)	45.4 (99)	42.4 (28)	p<0.001	p<0.001	0.778
Hyper convex nails on hands	17.7 (44)	26.1 (74)	27.1 (59)	22.7 (15)	0.022	0.019	0.526
Broad nasal bridge	8.8 (22)	23.2 (66)	24.3 (53)	19.7 (13)	p<0.001	p<0.001	0.508
Thin upper lip	10.4 (26)	21.8 (62)	23.4 (51)	16.7 (11)	p<0.001	p<0.001	0.308
Adherent ear lobes	13.7 (34)	20.4 (58)	17.9 (39)	28.8 (19)	0.050	0.250	0.080
Asymmetric face	0.0 (0)	19.0 (54)	17.0 (37)	25.8 (17)	p<0.001	p<0.001	0.151
Fused eyebrows (synophrys)	6.8 (17)	16.2 (46)	11.9 (26)	30.3 (20)	0.001	0.077	0.001
Epicanthus	40.6 (101)	13.0 (37)	12.4 (27)	15.2 (10)	p<0.001	p<0.001	0.537
Retarded toe (fourth or fifth)	4.0 (10)	10.9 (31)	11.0 (24)	10.6 (7)	0.003	0.004	1.000

CoF Control Families; CaF Case Families; (CaF w/P) Case Families without Patient; P Patient with schizophrenia

The concordance of the MPA was only shown to be significant (>0.30) in four anomalies: overlap of the toes, clinodactyly, narrow and high palate and fine electric hair. Table 4.

**Table 4:** Concordance between kinship and group.

	Case Families			Control Families		
Anomaly	Father Offspring	Mother Offspring	Between Brothers	Father Offspring	Mother Offspring	Between Brothers
Curved fifth finger (Clinodactyly)	56.9%	66.9%	58.7%	34.6%	39.4%	40.8%
Fine electric hair	45.0%	37.5%	35.0%	20.5%	21.3%	8.5%
Overlapping toes (any pair)	30.6%	25.6%	21.0%	43.3%	39.4%	56.3%
High / steeples palate	27.6%	32.3%	36.6%	35.6%	45.6%	33.6%

The heritability for the total MPA was 34.9 for the case families and 54 for the control families [F(1,121) = 5.00, p = 0.027]

## Discussion

In this study, the total number of MPA was higher in the members of families with a member with schizophrenia compared to the members of control families; the difference was 1.26 anomalies, representing a magnitude of effect size of d = 0.58. There is no reference in the literature, as this study is the first to compare the members of complete nuclear families. The effect size between patients and healthy nonfamily subjects in this study was d = 0.77, which is considered a medium effect, and this value contrasts with the value found between patients and controls in the published meta-analysis, where the combined magnitude of the effect size was d = 1.13 in the study by Weinberg and d = 0.95 in the study by Xu. The smaller magnitude of the effect size is explained by the larger sample size.

The members of the control families differed from the first-degree family members of patients by 1.13 anomalies with d = 0.52. Regarding studies that compare the unaffected family members of patients with healthy subjects, in the meta-analysis of Xu, a nonsignificant d = 0.32 was obtained based on six items, of which four do not present differences and two of them do. In this study, the differences were significant, supporting the susceptibility of the family members of patients with schizophrenia to present an increased number of MPA.

Patients compared with their family members showed a nonsignificant difference of 0.55 anomalies with d = 0.10, in contrast to the results presented by Xu, where a combined six studies found differences. This study is consistent with three others not included in the meta-analysis [31–33] The differences by anomalies are distinct to the study and demonstrate heterogeneity.

In summary, the members of the case families differed from the members of the control families; the patients did not differ from their family members; and the members of control families differed from the members of the case families excluding the affected family members. As explained previously, these results are consistent with the literature, which allows us to infer that MPA are present more frequently in patients with schizophrenia, followed by their family members. It is important to note that the Gourion Scale used includes 41 anomalies and that the average differences are of less than two anomalies.

In the control families, the heritability of the total score of the scale is 54%. It is believed that between parents and offspring, the theoretical heritability is 50%. This discrepancy between heritability in case families compared to control families is influenced by the lower number of anomalies in the controls.

The association between each anomaly indicates that only eleven anomalies presented the suggestive pattern of familial aggregation. Of these eleven anomalies, ten are reported in the literature (clinodactyly, Big gap between first and second toes (sandal gap), overlap of the toes, hyperconvex nails on the hands, the basis of an abnormally large nose, thin upper lip, malformed ears, significant asymmetry of the face, an abnormally short toe); this study is consistent with other studies [34–35], noting that these other studies only compare patients with healthy subjects. The tapered fingers in the hands present the same pattern, but the literature does not report this anomaly as significant.

Concordance of the MPA among the members of case families appeared in only three anomalies: clinodactyly, fine electric hair and overlap of the toes. With moderate values among members of the control families, the epicanthus also appears concordant. Consistency between first-degree family members is a measure of heritability, and considering that 41 anomalies were evaluated, there are very few that could have a genetic component, supporting the neurodevelopmental theory.

There are differences that suggest familial aggregation in the total score of the scale, but its use as an endophenotype is not relevant because it does not comply with the criteria of Gottesman [36] and Waldman [37]. As Ambrosio [38] reported the Gourion Scale for the studied sample does not present internal consistency or construct validity.

An MPA scale that represents an endophenotype should consist of a list of MPA with proven genetic components instead of MPA that demonstrate heterochrony of development, which could have led to the development of minor malformations, deformations or changes [39].

This scale is only appropriate as a biological marker of neurodevelopment instead of an endophenotype. Familial aggregation is confirmed at the level of the sum of MPA but not in each individual MPA, supporting that schizophrenia has a foundation in the neurodevelopmental theory. Given the above, it is proposed that it would be necessary to look for a genetic susceptibility to suffering congenital accidents.

The strength of this study lies in the fact that the samples (cases and controls) correspond to complete nuclear families (including a father, mother and offspring), in addition to the fact that the sample considered incorporates a larger number of subjects, and no other study has this type of coverage.

## Supporting Information

S1 File Data file. (XLSX)
